# IgG4 Disease-Related Ataxia

**DOI:** 10.1007/s12311-023-01592-8

**Published:** 2023-08-09

**Authors:** Marios Hadjivassiliou, Daniel Blackburn, Ronan O’Malley, Nigel Hoggard

**Affiliations:** 1 Academic Department of Neurosciences, Royal Hallamshire Hospital, Glossop Road, Sheffield, S10 2JF UK; 2 Department of Infection, Immunity and Cardiovascular Disease, University of Sheffield, Sheffield, UK

**Keywords:** IgG4 disease, Cerebellar ataxia, Behavioural frontotemporal dementia, Large vessel vasculitis

## Abstract

We describe a male patient presenting with cerebellar ataxia and behavioural frontotemporal dementia in whom imaging showed cerebellar atrophy. He had significantly low N-acetyl aspartate to creatine (NAA/Cr) area ratio on MR spectroscopy of the cerebellum, primarily affecting the vermis. CT body scan showed extensive abnormal tissue within the mesentery, the retroperitoneum and perinephric areas. PET-CT showed increased tracer uptake within the wall of the aorta suggestive of an aortitis and within the perinephric tissue bilaterally. Biopsy of the perinephric tissue confirmed IgG4 disease. Treatment with steroids and mycophenolate improved his clinical state, but he developed symptoms attributed to pericardiac effusion that necessitated treatment initially with drainage and subsequently with pericardial window. After a course of rituximab, he had an episode of sepsis that did not respond to appropriate treatment and died as a result. Both the imaging findings and neurological presentation with cerebellar ataxia and behavioural frontotemporal dementia are novel in the context of IgG4 disease.

## Introduction

IgG4 disease is a chronic inflammatory condition characterised by tissue infiltration with lymphocytes and IgG4-secreting plasma cells [1]. It results in fibrosis and tissue destruction. Depending on the site of the tissue infiltration, IgG4 disease can present as a consequence of obstruction/compression, e.g., jaundice due to the involvement of the pancreas, biliary tree and urinary symptoms and hydronephrosis due to the involvement of the urinary tract [2, 3]. It can also present as a consequence of mass effect as is the case in Riedel’s thyroiditis, retroperitoneal fibrosis and interstitial nephritis. A number of well-recognised disease entities, previously of unknown aetiology, are now attributed to IgG4 disease including autoimmune pancreatitis, sclerosing cholangitis and sialadenitis (Mikulicz’s disease) [4]. IgG4 disease has also been linked to arterial inflammation including aortitis and large vessel vasculitis [5]. In the revised Chapel Hill Consensus Conference on vasculitides, IgG4 disease has been listed as a cause of large vessel vasculitis [6].

Neurological involvement can take the form of pachymeningitis manifesting with headaches and/or cranial nerve involvement [7]. It is very likely that a substantial number of patients labelled as having “idiopathic” pachymeningitis have IgG4 disease. Diagnosis relies on review of biopsy tissue with special staining for IgG4-secreting plasma cells. Pituitary involvement can also be seen as part of pachymeningitis or in isolation. IgG4 disease can be clinically indistinguishable from neurosarcoidosis, although histological examination should be sufficient to distinguish between the two provided that IgG4 disease is specifically looked for.

A novel neurological presentation with pure cerebellar ataxia has previously been described in the context of IgG4 disease by our group [8]. This was a patient with large vessel vasculitis presenting with pure cerebellar ataxia and brain imaging showing involvement of the cerebellar peduncles. The patient had raised serum levels of IgG4, but no biopsy was possible to confirm the diagnosis. He responded well to steroids and mycophenolate. Here, we describe another case of IgG4 disease, this time biopsy proven, presenting with cerebellar ataxia and cognitive dysfunction.

## Case Report

A 67-year-old man was referred to neurology because of altered personality and disinhibited behaviour. Over the last 6 weeks, the family noticed slurred speech. He had become much more impulsive, agitated and easily irritated. He could be verbally aggressive and made inappropriate comments about people’s weight and ethnicity in public spaces. He admitted symptoms of loss of balance and excessive drinking of water starting 1 year before. There was a past history of trigeminal neuralgia and polymyalgia rheumatica, but he was on no medication.

Examination demonstrated no nystagmus, normal saccades, slurring of speech with arm dysmetria and dysdiadochokinesia as well as heel to shin and gait ataxia. Tone was normal. He was able to walk unaided but could not tandem walk or stand on each leg in turn. There was no weakness or sensory disturbance. Reflexes were present and symmetrical. There was an evidence of ideomotor and constructional apraxia. He had limited insight of his problems but was aware of the unsteadiness of gait. A formal neuropsychological assessment identified a profile consistent with behavioural variant of frontotemporal dementia. He exhibited difficulties with response inhibition, cognitive flexibility, verbal abstract reasoning and strategic reasoning and planning. Language and visuo-construction was spared, but there were deficiencies in planning and visual organisation. During the testing, he was observed to be disinhibited and impulsive. There was increase in apathy, disinhibition and executive dysfunction.

Initial investigations demonstrated elevated erythrocyte sedimentation rate at 105 mm/hr (normal range 1–10), C-reactive protein of 28 (0–5 mg/L), high platelet count at 508 (150–400x10^9^/L) and raised IgA and IgM immunoglobulins with the presence of IgG kappa monoclonal immunoglobulin of 2.7 g/L. Previous bone marrow examination resulted in the diagnosis of monoclonal gammopathy of undetermined significance. Renal, liver and thyroid function tests were normal. Immunological screen including antinuclear antibodies, double-stranded DNA, extractable nuclear antigens, anti-GAD, antinuclear cytoplasmic antibodies, thyroid antibodies, coeliac serology and paraneoplastic antibodies were all normal.

**Fig. 1:**
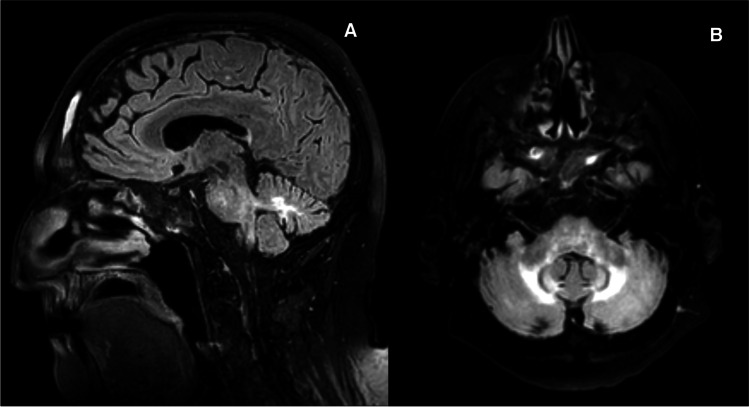
Magnetic resonance imaging (FLAIR sequence) showing increased signal in the deep white matter (**A** sagittal, **B** axial) of both cerebellar hemispheres with some less prominent involvement of the cerebellar peduncles and brainstem

Brain imaging showed mild cerebellar atrophy and increased T2-weighted signal within the deep cerebellar hemisphere white matter (Fig. 1). The posterior pituitary gland T1-weighted bright spot was absent on the pre-contrast imaging, but the pituitary gland was otherwise normal. Such appearance can be seen in the context of diabetes insipidus.

Cerebrospinal fluid examination showed no cells, normal glucose and protein.

CT body scan showed extensive abnormal tissue within the mesentery, the retroperitoneum and perinephric areas. The perinephric tissue was also involving the proximal ureters resulting in bilateral hydronephrosis for which monitoring of the renal function was recommended. An echocardiogram showed good LV function and moderate pericardial effusion.

Endocrine review diagnosed diabetes insipidus treated with Desmopressin nasal spray. The remaining pituitary function tests were normal.

MR spectroscopy of the cerebellum showed profoundly reduced N-acetyl aspartate to creatine (NAA/Cr) area ratio from the vermis (0.58, normal > 1) with well-preserved hemispheric ratio at 1.03.

A whole body FDG-PET scan showed increased tracer uptake within the wall of the aorta suggestive of an aortitis. This extended throughout the length of the aorta. Intense tracer uptake was also seen within the perinephric tissue bilaterally.

A CT-guided biopsy from the perinephric tissue showed patchy dense lymphoplasmacytic infiltration and fibrotic stroma with thick collagen bundles. Immunohistochemistry for CD38 highlighted IgG4 positive plasma cells > 10/high-powered field. The findings were in keeping with IgG4 disease. The patient was treated with oral steroids and subsequently mycophenolate. Initially (first 3 months), there was a gradual improvement in his ataxia and cognitive state.

The patient complained of shortness of breath worsen by minimal exercise. Investigations revealed worsening pericardiac effusion. The steroid dose was increased, and he had the effusion drained. A decision was made to treat with rituximab. He developed hypotension. Further imaging confirmed recurrence of the pericardiac infusion, and concerns were raised about pericardiac tamponade. He underwent pericardial window procedure at the cardiothoracic unit and initially responded well. Histological examination of the pericardial tissue confirmed fibrotic changes with some inflammatory cells in keeping with IgG4 disease. He received the first dose of a course of rituximab.

He was transferred back to the district general hospital with arrangements to be discharged home. Whilst an inpatient, he became suddenly unwell due to sepsis of unknown origin. Despite antibiotics and supportive care, he passed away shortly after.

## Discussion

We report a novel case of IgG4 disease presenting with cerebellar ataxia and behavioural frontotemporal dementia. Additional features included aortitis with raised inflammatory markers but complete absence of headache. It is unclear if at least some of the cognitive problems were related to cerebellar cognitive affective syndrome or if this was directly related to the IgG4 disease affecting other parts of the brain. Certainly the neuropsychological assessment suggested a more extensive involvement.

In keeping with what is often seen in other immune-mediated ataxias, both clinically and on MR spectroscopy, there was preferential involvement of the cerebellar vermis with corresponding gait ataxia and profoundly reduced NAA/Cr on spectroscopy of the vermis [9]. In addition, there were some unusual changes on MR imaging of the cerebellum showing increased T2-weighted signal within the deep cerebellar hemisphere white matter (Fig. 1). The aetiology of these changes remains unclear. The very symmetrical changes and position argue against an ischaemic aetiology related to the large vessel vasculitis. An immune-mediated explanation remains most likely. Symmetrical changes confined to the cerebellar peduncles can be seen in cerebellar variant of multi-system atrophy and in fragile-X tremor ataxia syndrome. The changes seen here, however, were much more extensive and also involved the deep cerebellar hemisphere white matter. Furthermore, the overall clinical picture was not suggestive of these 2 conditions.

Although IgG4-related disease was described two decades ago, the lack of familiarity and diverse clinical presentations means that a large proportion of patients remain undiagnosed. The diverse presentation is a result of involvement of many different organs in combination or in isolation. Biopsy is essential to make the diagnosis as many of these patients may have normal serological levels of IgG4 as was the case here. Whilst in the majority of cases accessibility to tissue for biopsy is possible, in patients presenting with neurological manifestations in isolation, this can be problematic, apart perhaps from patients presenting with pachymeningitis. The patient described here, however, had additional involvement with retroperitoneal, perinephric and pericardial abnormalities.

The overall immunopathogenesis of this disease remains unclear, but the pathogenic role of B cells and humoral immunity is beginning to unravel. Oligoclonal expansion of IgG clones has been shown to be present in the peripheral blood of patients [10]. These same clones were identified in the tissue involved. Treatment with steroids causes reduction in the circulating clones in conjunction with clinical improvement. The role of the IgG4 molecule remains obscure. There is a correlation between the concentration of IgG4 and the severity of organ involvement as well as risk of relapse. Some work on autoantigens in the context of autoimmune pancreatitis due to IgG4-related disease has revealed that half of such patients had antibodies against laminin-511 [11]. Immunisation of mice with human laminin-511 protein was shown to lead to the development of antibody responses resulting in pancreatic injury [11]. This observation supports the concept that autoantigens contribute to the pathophysiology of IgG4-related disease.

The first-line treatment of IgG4 disease is steroids which are effective in the vast majority of patients [12]. However, up to 40% of patients may fail to achieve complete remission or may relapse within a year. Low-dose cyclophosphamide and mycophenolate have been shown to significantly reduce the rate of relapse. Rituximab usually has a dramatic effect, often with reduction in size if not complete resolution of the tumour like masses. Unfortunately, our patient developed sepsis, a known complication of immunosuppression and died as a result.

We conclude that cerebellar ataxia with or without cognitive deficits should be added to the list of neurological manifestations of IgG4 disease.

## Data Availability

Not applicable.
